# Correlation between religiosity, family functioning, and factors associated with substance use among secondary school students in high-risk areas

**DOI:** 10.1371/journal.pone.0308192

**Published:** 2025-07-10

**Authors:** Noor Adnin Ab Aziz, Suzaily Wahab, Rosnah Sutan, Muhammad Adib Baharom, Amirul Danial Azmi, Siti Azirah Asmai

**Affiliations:** 1 Department of Psychiatry, Faculty of Medicine, Universiti Kebangsaan Malaysia, Kuala Lumpur, Malaysia; 2 Department of Public Health Medicine, Faculty of Medicine, Universiti Kebangsaan Malaysia, Kuala Lumpur, Malaysia; 3 Department of Psychiatry, Hospital Selayang, Selangor, Malaysia; 4 Department of Computer Science, Faculty of Information and Communication Technology, Technical University of Malaysia, Malacca, Malaysia; University of the West Indies at Saint Augustine, TRINIDAD AND TOBAGO

## Abstract

**Introduction:**

Substance use in adolescents poses a complex societal challenge, undermining nation-building and socioeconomic growth. Religiosity encompasses a person’s religious beliefs, habits, and involvement in spiritual activities. Family functioning refers to a family unit’s overall health and operation, including communication, emotional bonding, support, roles, and behavioral control. Both aspects are pivotal in determining substance use in adolescents. This study assesses the association between religiosity and family functioning and determines factors concerning substance use among adolescents in secondary schools in high-risk areas.

**Methods:**

A cross-sectional study was conducted with 312 adolescents from selected secondary schools in substance use hotspot areas in Northern Malaysia. The tools used were Alcohol, Smoking, and Substance Involvement Screening Tool-Lite (ASSIST-Lite), Family Adaptation and Cohesion Scale version IV (FACES-IV), and Hatta Islamic Religiosity Scale (HIRS).

**Results:**

The prevalence of substance use among adolescents was 9.6% (n = 30). Most users used a single substance (76.7%; n = 23), and only 23.3% (n = 7) used multiple substances. The mean age was 14.13 years (SD:0.67), the majority being Malays (99.0%; n = 309) with an Islamic background. Adolescent substance use was significantly associated with gender (16.3% in males and 6.3% in females, P = 0.004) and having a recent family history of substance use (16.8%, P = 0.003). No significant association between substance use and religiosity scores were elicited (W(1)=2.610, P = 0.106 and W(1)=0.092, P = 0.761 for knowledge and practices, respectively). However, substance use was found to be associated with family functioning subscales, which were chaotic family scores (W(1)=4.588, P = 0.032), and family satisfaction score (W(1)=4.831, P = 0.028). Regression analysis revealed that being male (Adj.OR=3.08, P = 0.006) and having a recent family history of substance use (Adj.OR=3.17, P = 0.004) significantly predicted substance use.

**Conclusions:**

This study highlights the role of chaotic family dynamics and family satisfaction and its influence onto adolescent substance use. Despite the insignificant finding between religiosity and substance use, further exploration in this area is may prove beneficial to enhance care for individuals.

## Introduction

Substance use is a worldwide public health problem with a rising trend among adolescents [1,2]. The World Drug Report 2023 revealed that in 2021, one in seventeen people aged between 15 and 64 had used a substance within the past year worldwide [3]. Substance use refers to the consumption or intake of psychoactive substances, including tobacco, alcohol, prescription medications, and other illicit substances. Tobacco and alcohol are examples of licit psychoactive substances. Amphetamine-type stimulants, heroin, cocaine, marijuana, and lysergic diethylamide (LSD) exemplify illicit substances. Substance use has a substantial long-term impact on public, the criminal justice, and health care services [4]. In Malaysia, the National Anti-Drugs Agency (NADA) under the Ministry of Home Affairs reported that from 2016 to 2020, a rise occurred in the number of substance use cases detected by the state, along with a rise in use among adolescents. The report also underscores that synthetic drug use was more prevalent than organic drugs, predominantly methamphetamine (crystalline and pills) and amphetamine-type stimulants use [5].

Religiosity, defined as the extent and intensity of a person’s religious beliefs, practices, and experiences, notably varies among cultures [6]. It is pivotal in shaping adolescents’ behavior and lifestyle choices (including substance use). In Western countries, religiosity is often portrayed as personal and individualized, with a high degree of secularism and various religious affiliations. Differently, religiosity in Southeast Asian countries is mainly shown as something that is communally practiced and is profoundly incorporated into daily life. In Malaysia, for instance, Islam is a state-endorsed religion that the major racial group, the Malays, practice [7].

Previous research has demonstrated the link between religiosity, family functioning, and substance use. Numerous studies have illustrated that religiosity is a noteworthy protective factor against substance use [8–11]. This protection is possibly due to an indirect effect on self-control via perceived immorality acquired from stronger religiosity [12]. Some local studies have managed to replicate similar findings, accentuating that Malaysian youths with solid religious views were more protected against substance use [13,14]. However, the National Anti-Drugs Agency has reported that an overwhelming majority of adolescents who use substances have an Islamic background [5]. This situation poses the question of whether religiosity truly impacts these adolescents’ substance use in Malaysia.

Furthermore, family functioning is an equally important factor in adolescent substance use and has been demonstrated to affect a person’s physical and psychological development profoundly. Family functioning refers to the quality of interactions and relationships within a family unit. A family unit’s primary functions include instilling beliefs, values, and acceptable behavior in society [15]. Previous studies have revealed that inadequate family functioning contributes to substance use [16–18]. As Wills and Yaeger (2003) state, factors such as good familial support and communication protect people from substance use. In contrast, adverse factors like parent-child conflict result in inferior self-control on the adolescent’s part [19]. This circumstance cascades into possible negative life events and deviant peer affiliations, predisposing these adolescents to further possibilities of substance use.

A complex interplay exists between family functioning and religiosity with substance use as both factors play a protective role, directly or indirectly, against substance use. Furthermore, family functioning and religiosity are correlated, possibly affecting substance use. Adolescents with superior relationships with their parents display more profound religiosity (concerning belief and practice) than those with inferior relationships [20,21]. Conversely, a strain in the adolescent-parent relationship might occur if they do not share similar religious views or beliefs, fostering adolescent delinquent behavior [22]. Another study conducted amongst adolescents discovered that religiosity may be pivotal in mediating family functioning, especially regarding parental monitoring and setting boundaries and in reducing substance use [23]. This was also suggested in an older study where it was found that religiosity was the most potent mediator between family support and change in alcohol consumption behavior [21].

In 2019, NADA identified 155 substance-related hotspots in Malaysia, where the northern region (particularly the areas bordering Thailand) was a critical hub for substance use [24,25]. Adolescents in these hotspot areas may have increased exposure to substance use than those in other areas.

People are vulnerable to substance use during adolescence because this period is marked by a high tendency toward seeking new experiences, increased susceptibility to peer pressure, and decreased self-esteem [26]. Substance use often begins between 12 and 17 years old, with its use peaking between 18 and 25 years old [27]. Early engagement in substance use elevates the chances of developing substance use disorder. Furthermore, using licit substances, tobacco and alcohol, predisposes them to other illicit substances [28]. This issue is aggravated further because the deleterious effects of substance use may remain in adolescents far into their adulthood [29]. A recent nationwide survey in Malaysia has reported several alarming findings where roughly 105,000 adolescents were reported to have tried substances at least once in their lives. Moreover, about 60% of them still actively use them, highlighting a need to investigate the causes and possible protective factors against substance use holistically [30].

Given the numerous factors contributing to adolescent substance use and the lack of local studies addressing adolescents in hotspot areas, we aim to explore the relationship between substance use, religiosity, and family functioning among adolescents. We hypothesize that religiosity and family functioning are associated with substance use among adolescents in Malaysia.

## Methodology

### Research design, study area, and data collection

A cross-sectional study was conducted in selected secondary schools in North-Eastern Peninsular Malaysia. Based on the data from NADA, three hotspot districts from one state were identified, and two schools from each district were chosen. The school names were not disclosed due to potential sensitivities as the study addressed substance use among adolescents.

Data were obtained using stratified random sampling from December 1st to 15th, 2023. Class teachers and students were informed about the study’s objective, methods, benefits, and risks, assured anonymity, and the withdrawal option from the study at any point. Only those with consent from both parents and who have given assent themselves were recruited to complete the questionnaires using Google Forms, an online survey platform. The questionnaires include items on sociodemographic factors, religiosity levels, substance use risk, and family functioning scores. The respondents were allowed to answer the questionnaires at their own pace and time to help ensure a more accurate response. The online method of data collection also allows privacy for the respondents as the items in the questionnaire are of a sensitive topic. The Institution’s Research Ethics Committee (UKM PPI/111/8/JEP-2023-076) approved the study and permission to conduct it with school students was received from the Education Planning and Research Division, Ministry of Education Malaysia.

The sample size was calculated using the formula N = Z^2^P(1-P)/d2 [31]. With the reported prevalence of 17.2% from a previous study [18] and a precision of 0.05, the required sample size was 215. Forty-four additional respondents were included to account for a 20% non-response rate, bringing the sample size to 263.

The inclusion requirements included being a school student enrolled in a public secondary school, having an Islamic background, and an understanding and ability to write in Malay or English. The study excluded 17-year-olds since as they were among those who would be sitting for a major exam.

### Study instruments

All respondents were asked to complete a sociodemographic questionnaire, the Alcohol, Smoking, and Substance Involvement Screening Tool-Lite (ASSIST-Lite), the Hatta Islamic Religiosity Scale (HIRS), and the Family Adaptation, and Cohesion Scale Version IV (FACES-IV). All questionnaires were already translated into the Malay language and validated. Permission to use the questionnaires was obtained from the original authors and Cronbach alpha was assessed based on our targeted samples to quantify the internal consistency and reliability of items. Values between 0.7 and 0.9 are considered good indications of internal stability of a tool.

The sociodemographic, HIRS, and FACES-IV questionnaires were used to ascertain the independent variables, which were age, gender, race, total household income, past family history of substance use, parents’ highest education level, religiosity scores, and family functioning scores. Whereas ASSIST-Lite was used to measure the substance use profile of the respondents as the dependent variable.

### Alcohol, Smoking, and Substance Involvement Screening Tool-Lite (ASSIST-Lite)

Substance use was determined using a validated Malay version of ASSIST-Lite, an abbreviated version of ASSIST. It is a screening tool to assess the substance use, the frequency of usage, and the use of various substances of concern, such as tobacco, alcohol, cannabis, amphetamine-type stimulants, sedatives, opiates, and others within the last 3 months. The instrument, comprising six components with 3–4 items and two additional questions, was translated and validated for use in Malaysia, totaling to 21 items. Both internal and test-retest reliability were strong, with scores ranging from 0.772 to 0.882 and Kappa values from 0.8 to 1 [32]. Internal reliability was performed among current targeted samples who were recruited among 13–16 years old students enrolled in a secondary school in a northern state in Malaysia. The ASSIST-Lite scored an internal reliability score range of 0.370–0.785 for all the domains. The internal reliability score for the questionnaire (except cigarette use) was low and this may be partly due to the low number of participants having had used these substances (cigarettes (n = 15), alcohol (n = 7), cannabis (n = 5), ATS (n = 4), hallucinogens (n = 4), and opiates (n = 3)).

### The Hatta Islamic Religiosity Scale (HIRS)

The HIRS is a 27-item scale developed by Mohamed Hatta Shaharom in 1996 to assess fundamental Islamic knowledge and practice among Muslim adults and adolescents in Malaysia. These two domains have also been previously measured in previous Islamic religiosity studies [33,34]. The scale was written in Malay and comprises four parts: 15 questions on fundamental Islamic knowledge based on the primary and lower secondary school curriculum, ten questions on fundamental Islamic practices required of practicing Muslims according to the Quran and Hadith, one question on the frequency of Qur’an reading, and one question evaluating the respondent’s efforts in enjoining the good and forbidding the evil (bad habits), which are obligatory for all Muslims. Each component uses a Likert scale. Furthermore, a previous study had used the scale measuring religiosity of Muslim adolescents in Malaysian secondary schools and reported moderate levels of religiosity in this population [35].

The scale demonstrated high inter-rater reliability for the total HIRS96 score (Cronbach’s alpha range 0.81–0.90) in two independent reliability studies which were conducted with 286 respondents. The scale also demonstrated validity by successfully distinguishing between the religiosity levels of two groups—tahfiz students and delinquent youths (P < 0.05). A higher score indicates a higher level of religiosity [36]. In this study, the questionnaire was found to have an acceptable range of internal reliability score which were 0.687 and 0.533 for the Knowledge and Practice domains, respectively.

### Family Adaptation and Cohesion Scale Version IV (FACES-IV)

The FACES-IV was used to assess components of family functioning based on the Circumplex Model. It is a self-rated test with 42 items for balanced and unbalanced scales, ten for family communication, and ten for family satisfaction. The reliability of FACES-IV ranged from 0.77 to 0.89 across all six domains, with a high discriminant validity of 0.84–0.99 in all domains. The scale measured three aspects of family behavior: cohesion, adaptability, and communication. Cohesion demonstrates the emotional bond among family members, flexibility depicts changes in family leadership, roles, and rules, and communication helps families adjust their levels of cohesion and flexibility.

We used the Malay-translated version which underwent a back-to-back translation process with four psychiatrists who were fluent in both Malay and English, following the methods described by Sousa and Rojjanasrirat (2011) [37]. This translated version was pre-tested with 50 respondents. The Cronbach’s alpha for the translated version was 0.68. The original scale’s reliability coefficients ranged from 0.77 to 0.89, and the Polish version depicted Cronbach’s alpha values from 0.70 to 0.93, indicating good internal consistency. These values met the standards set by Olson and Gorall (2006), making the scale suitable for the research. FACES-IV was found to have good internal reliability with scores ranging from 0.712–0.892 for the balanced, unbalanced, family communication, and family satisfaction domains.

### Statistical analysis

The data were analyzed using the latest Statistical Package for Social Science (SPSS 29; SPSS Inc., Chicago, Illinois, United States). Categorical variables such as gender, age, race, total household income per month, and parents’ education levels were presented as frequencies and percentages, and the association between them was analyzed using the Chi-square test. Descriptive analysis determined the prevalence of substance use among secondary school students in high-risk areas. A simple logistic regression was conducted to analyze the association between the independent variables and the dependent variable (substance use). Finally, a binary logistic regression model was tested to identify factors predicting substance use (yes/no) among these students. Assumptions for the binary logistic regression model such as multicollinearity and logit correlation were tested prior to conducting the test.

## Results

### Analysis of respondents’ profile and substance use

Three hundred and twelve adolescents were selected from secondary schools to participate in the study. Table 1 provides an overview of the respondents’ sociodemographic characteristics and their substance use. The students’ ages ranged from 13 to 16, with 208 (66.7%) female and 104 (33.3%) males. The mean age of the students was 14.13 years (SD = 0.673). Almost all students were Malay (n = 309, 99.7%).

**Table 1:** Sociodemographic characteristics of participants and their substance use.

Variables	Total Number of Participants, n = 312 (%)	Substance Use		Chi-square χ² Value (d.f.)	P-Score
		Not Using, n = 282 (%)	Using, n = 30 (%)		
Gender*				8.132 (1)	0.004
Female	208 (66.7)	195 (93.8)	13 (6.2)		
Male	104 (33.3)	87 (83.7)	17 (16.3)		
**Total household income**				0.179 (1)	0.672
<RM4,850.00	262 (84.0)	236 (90.1)	26 (9.9)		
≥ RM4,850.00	50 (16.0)	46 (92.0)	4 (8.00)		
**Highest level of education for mother**				1.402 (1)	0.236
Up to secondary	232 (74.4)	207 (89.2)	25 (10.8)		
≥ Tertiary	80 (25.6)	75 (93.8)	5 (6.3)		
**Highest level of education for father**				0.839 (1)	0.360
Up to secondary	239 (76.6)	214 (89.5)	25 (10.5)		
≥ Tertiary	73 (23.4)	68 (93.2)	5 (6.8)		
Any family members recently used a substance*				8.949 (1)	0.003
Yes	101 (32.4)	198 (93.8)	17 (6.2)		
No	211 (67.6)	84 (83.2)	13 (16.8)		

*Significance at P-score < 0.05

Early adolescent (13–14 years old), Middle adolescent (15–16 years old), Total household income is based on the selected state’s median household income

Additionally, 262 (84.0%) came from lower-income families with a monthly household income of less than RM4,850.00 which was lower than the selected state’s median household income. Most had mothers (n = 232, 74.4%) and fathers (n = 239, 76.6%) with at least a secondary school level education. About a third, 101 (32.4%), reported a family member’s recent use of substances.

Of the 312 secondary school students, 30 (9.6%) reported recent single or polysubstance use. Most individuals who recently had used substances were in their early adolescence (n = 28, 93.3%), male (n = 17, 56.7%), coming from lower-income families with a monthly household income of less than RM4,850.00 per month (n = 26, 86.7%). Additionally, more than half, n = 17 (56.7%), reported a recent family history of substance use.

**Fig 1:**
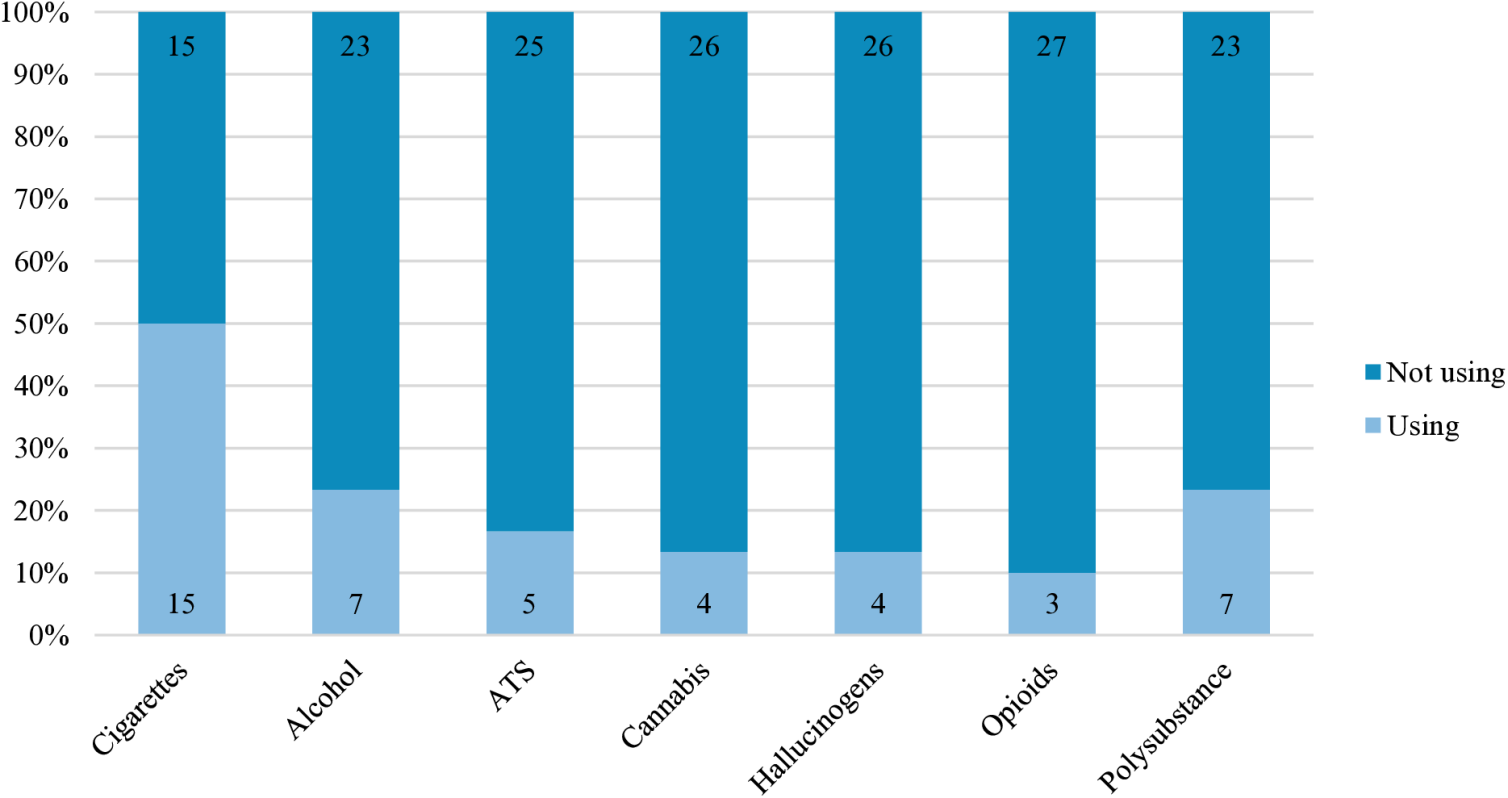
Types of substance used and substance use profile of the study population who uses substances (n = 30). Numbers within the bar chart indicates the number of samples using that substance.

Tobacco was the most widely used substance among the students (n = 15, 4.8%), followed by alcohol (n = 7, 2.2%). Stimulants (n = 5, 1.6%) were the most frequently used illicit substances. Among the students using substances, most used a single type of substance (n = 23, 76.7%). Fig 1 shows the breakdown of all the different substance type used and the substance use profile of the study respondents.

### Analysis of religiosity and family functioning

The Cronbach’s alpha values for the Hatta Islamic Religiosity Scale (HIRS) was between 0.55 and 0.69 for both the Islamic Knowledge and Islamic Practice subscales. The Family Adaptation and Cohesion Scale Version Four (FACES-IV) was also found to be statistically acceptable for this study population with the balanced, unbalanced, family satisfaction, and family communication scores having a Cronbach’s alpha value of 0.72–0.89.

The religiosity profile of respondents, based on the HIRS, revealed a mean score of 63.35 (SD = 8.61), indicating a high level of religiosity. Table 2 displays the scores for Islamic knowledge and practices, with mean scores of 23.04 (SD = 2.88) and 35.58 (SD = 7.15).

**Table 2:** Descriptive analysis of religiosity and family functioning of respondents.

Dimensions	Mean (SD)	Median (IQR)	Min-Max
**Religiosity profile**			
HIRS Total Score	63.35 (8.61)	64.00 (54.00, 64.75)	38.00–84.00
HIRS Islamic Knowledge	23.04 (2.88)	23.00 (21.00, 25.00)	16.00–30.00
HIRS Islamic Practice	35.58 (7.15)	34.00 (30.00, 42.00)	14.00–50.00
**Family functioning profile**			
**Balanced scales**			
Cohesion	24.79 (3.35)	25.00 (23.00, 27.00)	8.0–35.0
Flexibility	27.21 (3.62)	28.00 (26.00, 29.00)	8.0–35.0
**Unbalanced scales**			
Disengaged	17.40 (3.64)	17.00 (15.00, 20.00)	8.0–31.0
Enmeshed	20.49 (2.53)	21.00 (19.00, 22.00)	7.0–29.0
Rigid	22.95 (3.38)	23.00 (21.00, 25.00)	7.0–32.0
Chaotic	15.70 (3.92)	16.00 (13.00, 18.00)	7.0–27.0
**Ratio scores**			
Cohesion ratio	1.32 (0.25)	1.32 (1.13, 1.47)	0.64–2.30
Flexibility ratio	1.44 (0.30)	1.41 (1.27, 1.58)	0.68–3.09
Total circumflex ratio	1.38 (0.24)	1.38 (1.25, 1.52)	0.72–2.38
**Family scales**			
Family communication	37.37 (6.33)	38.00 (34.00, 41.00)	10.0–50.0
Family satisfaction score	37.79 (6.18)	39.00 (34.00, 41.00)	10.0–50.0

Similarly, Table 2 depicts the respondents’ family functioning profiles concerning balanced scales (cohesion and flexibility), unbalanced scales (enmeshed, rigid, disengaged, and chaotic), family satisfaction, and family communication. Each scale was accordingly categorized. The mean scores for the balanced scales (26.00) were higher than those for the unbalanced scales (19.14). Meanwhile, mean family communication and family satisfaction scores were 37.37 (SD = 6.33) and 37.79 (SD = 6.18), respectively. The cohesion ratio [balanced cohesion/ (disengaged + enmeshed/ 2)], flexibility ratio [balanced flexibility/ (rigid + chaotic/ 2)], and total circumplex ratio (cohesion ratio + flexibility ratio/ 2) all exceeded 1, demonstrating balanced family functioning.

### Association between sociodemographic characteristics, religiosity, and family functioning with substance use

Upon Chi-square analysis, only two sociodemographic characteristics were found to have significant associations with substance use. They were gender (χ^2 ^= 8.132, d.f. = 1, P = 0.004), and having a family history of substance use (χ^2 ^= 8.949, d.f. = 1, P = 0.003). Whereas race, total household income, and parents’ highest education level were found not significantly associated with substance use.

We found no significant associations between religious profile scores and substance use (P > 0.05). However, significant associations were found between family functioning scores and substance use particularly family satisfaction score (Wald (d.f. = 1) = 4.831; P = 0.028), and chaotic family score (Wald (d.f. = 1) = 4.588; P = 0.032) (Table 3).

**Table 3:** Simple logistic regression of variables with history of substance use.

Variables	Wald (d.f.)	Unadjusted Odds Ratio (95% C.I.)	P-value
Age	0.058 (1)	0.93 (0.52, 1.66)	0.809
FACES IV – Cohesion	0.526 (1)	0.96 (0.86, 1.07)	0.468
FACES IV – Flexibility	1.934 (1)	0.93 (0.85, 1.03)	0.164
FACES IV – Disengaged	1.011 (1)	1.05 (0.95, 1.17)	0.315
FACES IV – Enmeshed	0.066 (1)	1.02 (0.88, 1.19)	0.797
FACES IV – Rigidity	0.099 (1)	1.02 (0.91, 1.14)	0.753
FACES IV – Chaotic*	4.588 (1)	1.11 (1.01, 1.22)	0.032
FACES IV – Family Communication	2.773 (1)	0.95 (0.90, 1.01)	0.096
FACES IV – Family Satisfaction*	4.831 (1)	0.94 (0.89, 0.99)	0.028
HIRS – Knowledge	2.610 (1)	0.90 (0.79, 1.02)	0.106
HIRS – Religious Practices	0.092 (1)	0.99 (0.94, 1.05)	0.761

*Significance at P-value < 0.05

### Multiple logistic regression analysis

We used a enter method binary logistic regression analysis to identify predictors of substance use. The dependent variable was coded as “Having used substances” and “Not having used substances” whereas the independent variables were selected from the variables that were significantly associated with substance use in previous analyses.

Multicollinearity testing found the FACES-IV scores for Family Communication to be highly correlated with three other variables (Pearson’s r > 0.7) and was removed from the final model.

Adolescent boys were 3.08 times more likely to use substances than teenage girls (CI = 1.39, 6.85), p < 0.05). Respondents with a recent family history of substance use were 3.17 times more likely to use substances than those without such a history (CI = 1.44, 6.99, p < 0.05). The Hosmer-Lemeshow test indicated the model to be a good fit for this study (Chi-square 9.870, p = 0.274). The model achieved a Nagelkerke R^2^ score of 0.147 (Table 4).

**Table 4:** Multivariate binary logistic regression for substance use.

Variables	Wald (d.f.)	Adjusted Odds Ratio (95% CI)	P-value
Gender*	7.656 (1)		0.006
Female		1.00	
Male		3.08 (1.39, 6.85)	
Family members using substances*	8.148 (1)		0.004
No		1.00	
Yes		3.17 (1.44, 6.99)	
FACES IV – Chaotic	0.796 (1)	1.05 (0.94, 1.17)	0.372
FACES IV – Family Satisfaction	3.581 (1)	0.94 (0.88, 1.00)	0.058

*Significance at P-value < 0.05

Enter method with Hosmer-Lemeshow Test

## Discussion

Identifying patterns of substance use among adolescents is pivotal for effective prevention, intervention, and harm reduction strategies. Our study explored the connection between religiosity, family functioning, and substance use among secondary school students in Northern Malaysia, a high-risk area.

The prevalence of substance use in this study was 9.6%, aligning with the findings of a study in Egypt on polysubstance use [38]. However, it was lower than those in other countries, such as Timor-Leste, the United States, Norway, and Iran [39–42]. These differences could be explained by the data collection method (an online survey), sample size, and respondent characteristics. Other studies encompassed high school and university students, covering a broader age range (16–40 years), which may have resulted in an elevated prevalence rate. Cultural differences and social customs may have also been critical factors.

Our study found that cigarettes (4.8%) were the most commonly used substance, followed by alcohol (2.2%), stimulants (1.6%), inhalants (1.3%), and cannabis (1.3%). This result aligns with those of local research, demonstrating tobacco and alcohol use among secondary school students (aged 15–18) at 6.6% and 2.4%, respectively [26]. De la Torre-Luque and colleagues reported that tobacco was the most often used substance among adolescents in Indonesia, Laos, Malaysia, Myanmar, the Philippines, and Thailand [43]. However, our study revealed lower tobacco and alcohol use than the findings from the National Health and Morbidity Survey (NHMS) for Adolescents in 2022, depicting 9.0% and 7.4%, respectively [30]. Moreover, our study reports a lower prevalence than that in other Muslim-majority or Southeast Asian countries [44–46]. Nonetheless, it is still worrying to see many adolescents smoking cigarettes despite the cigarette ban for those under 18. This could partly be due to these adolescents being able to purchase cigarettes from commercial sources, indicating a lax in the practice of this ban [47].

Our study depicted that a higher proportion of males (56.7%) and Malays (99.0%) among those who used substances even though we had more female respondents in our study, coinciding with the data from the National Antidrug Agency of Malaysia [5]. The NHMS for Adolescents in 2022 also revealed that three out of four adolescents using substances started using them before the age of 14 [30], indicating similarity with our findings where we found that early adolescents (13–14 years old) were more likely to use substances.

Adolescent substance use was significantly associated with family members who had used substances (P = 0.003). Our findings revealed that at least half of the adolescents who used substances had at least one family member with substance use in the past. This finding was consistent with other studies that described the family environment as a social factor in youth substance use [13,42,48–50]. We based our hypothesis on Albert Bandura’s social learning theory and Ajzen’s theory of planned behavior. Bandura suggested that social learning environments encompass both models and learners. Furthermore, Ajzen’s theory posits that the perceived normalization of substance-use in ones surrounding may pressure them into using substance as well. In this context, learners observed and imitated the actions of their models, either intentionally or unintentionally, possibly explaining why adolescents were more likely to use substances if their family members did so [51,52].

Even though the percentage of adolescents using substances was higher among those from families of lower income, our study found no significant association between coming from low-income families and substance use (P = 0.672). This finding contrasted with the past studies’ findings, which found a significant link between low-income families and adolescent substance use [53–55]. This discrepancy could be attributed to the other factors that may have influenced substance use in this population. Furthermore, a local study exploring the predictor factors reported that household income was not significant in predicting adolescent substance use [25]. Moreover, our study could not prove a significant association between parents’ education level and adolescent substance use (P = 0.236 and P = 0.360 for mother and father, respectively), unlike other studies demonstrating that an association existed between parental education level and substance use [13,53,54]. Parents with lower education levels may face several challenges in overcoming the issues of substance use, including a lower awareness of the dangers and economic challenges that may predispose these adolescents to resort to substances as a coping mechanism. This finding indicates that among the sample used in our study, other factors play a more critical role in influencing substance use in adolescents.

Our findings did not reveal any association between religiosity and substance use. This finding contrasted with those of other studies that have demonstrated a significant relationship between lower religiosity scores and substance use. These past studies have posed that with high religiosity, the reduced rates of substance use were possibly linked to having a much more conservative attitude against substance use and improved psychological well-being (which includes protection against suicidality) [11,56–59]. The differences could be contributed to the use of different questionnaires from these studies which may have assessed the different domains of religiosity. The questionnaire used in this study focuses solely on knowledge and attitudes towards religion. Religiosity is a multi-dimensional construct with nuances, particularly in the different effects of intrinsic (internalized personal beliefs and spiritual commitment) and extrinsic religiosity (social or cultural expectations of religious practices), yielding varying substance use outcomes. An Iranian study reported that internalized religious beliefs were significantly associated with lower perceived stress and nicotine addiction, differing from the extrinsic counterpart [60]. This finding was also reported in a previous study, where private (internalized) religiosity was a significant protective factor against sensation-seeking and tolerant views toward substance use. Thus, it acts as a buffer preventing possible substance use among adolescents [61]. Conversely, a study conducted in Türkiye suggested that a religious social environment (extrinsic religiosity) to be the bigger protective dimension against youth substance use when compared to belief and self religious practices (intrinsic religiosity) [62]. Furthermore, as highlighted by Salas-Wright et. al. (2014), adolescents who disapprove of substance use are more likely to refrain from substance use irrespective of religiosity. This could have been a potential factor that may have played a role in affecting adolescent substance use in this study.

Altogether the respondents in our study scored higher on the balanced family functioning (cohesion and flexibility) scale than the unbalanced family functioning (disengagement, enmeshment, chaos, and rigidity) scale. The samples in our study are reported to have good connection and flexibility levels of family functioning [55]. Family functioning concerning balanced flexibility, family satisfaction, and communication was negatively correlated with substance use, suggesting that high family flexibility, good communication, and high family satisfaction protect against substance use.

High family flexibility refers to the family’s ability to adjust to changes, manage stress, and reorganize roles. This adaptability created a stable and dynamic environment where adolescents felt supported and understood, lowering the likelihood of resorting to substances to handle stress or uncertainty. Effective family communication was pivotal in building trust, understanding, and emotional support. When family members communicated openly, adolescents would express concerns, seek advice, and feel valued. This open communication also assisted in detecting early signs of distress or harmful behaviors, allowing for timely interventions.

High family satisfaction reflected positive family experiences, leading to strong emotional connections, mutual support, and happiness. Adolescents who were satisfied with their family relationships would adopt positive family values and norms, discouraging them from seeking comfort or validation through substance use.

Our study also revealed a significant association between chaotic family environments and substance use. This study also found significant associations between family flexibility, family cohesion ratio, and circumplex ratio scores with substance use. Furthermore, family satisfaction and communication were also found to be significantly associated with substance use. Adolescents from unbalanced families would have an increased risk of substance use, aligning with decades of research accentuating the link between family functioning and substance use. Factors like parental psychopathology, family conflict, relational distance, parenting deficits, and family’s inability to adapt flexibly were predictors of substance initiation and abuse [16,63,64]. These variables were closely associated and could be the predisposing, precipitating, or perpetuating factors for both substance use and family conflict. Our findings aligned with previous research indicating that a healthy family dynamic and strong parent-adolescent relationships played a protective role against adolescent substance use [16,56,65–68]. In a study by Zeng & Tan (2022), it was found that individuals coming from functional families show better optimism and self-efficacy when it comes to reducing substance use relapse tendency [69]. This was in line with what was posited by Bandura, where both optimism and self-efficacy are important drivers impacting changes in behavior [51]. Similarly in line with Ajzen’s theory of perceived behavioral control, adolescents with dysfunctional families, and especially those from families who use substances, may lack the required ability of inhibition and self-control making them feel less empowered to resist using substances [70].

Our final analysis using logistic regression revealed that male gender, and recent family history of substance use significantly predicted substance use among adolescents. Our findings revealed that male adolescents have roughly 3 times higher odds than female adolescents to use substances. This was consistent with previous researches reporting higher substance use among males [38,42,48,53,64,71]. The odds of substance use was 3.17 times higher (CI: 1.11–6.43) for adolescents from families with a history of substance use compared to those from families who do not use any substances. This finding aligned with earlier studies posing that adolescents are more likely to use substances if family members do [49,72]. This study would like to highlight family satisfaction as a protective factor against substance use, as it attained a p-value close to conventional threshold of significance (P = 0.058). In this study, with every increase in family satisfaction score, adolescents have 0.06 times less odds to use substances or in other words, for every 10 points increase of the family satisfaction score, these adolescents have a 46.1 percent less odds of using substances. As measured by FACES-IV, family satisfaction determined how content adolescents were with their relationships and family dynamics, such as communication, emotional bonding, support, problem-solving, and adaptability. Higher scores indicated greater contentment and effective family functioning, whereas lower scores suggested detachment or dissatisfaction. Previous research also accentuated the protective role of family satisfaction against adolescent substance use [73].

In summary, religiosity was not a significant predictor of substance use among adolescents, contrasting the assumption that religious beliefs protect against risky behaviors. This finding suggests that religiosity alone may be inadequate to prevent substance use, or its impact depends on other factors like peer pressure or family environment. Conversely, being male, and having family history of substance use were significant predictors of adolescent substance use. This result suggests the need for gender-specific interventions, particularly for male adolescents, and targeted support for families with a history of substance use.

### Study limitations

Our study has some limitations. First, the study’s cross-sectional design limits its ability to establish causality because it only captures data at a single time, making it unclear whether changes in religiosity or family functioning influence substance use or vice versa. Furthermore, this design is prone to temporal ambiguity and potential confounding factors such as socioeconomic status or family history of substance use, affecting both the independent and dependent variables. Additionally, recall bias and social desirability bias could impact the accuracy of self-reported data from adolescents. Hence, the study’s findings may only be generalized within the sampled population. A longitudinal study design may prove beneficial in ascertaining the causality of religiosity or family functioning on adolescent substance use. Following a longitudinal study conducted by Mason & Windle (2001), it would be beneficial to also conduct a future study not just on alcohol consumption but also to the use of other substances [21].

The study has other limitations that limit its generalizability. Substance use data were obtained through self-reporting in this study. Thus, an objective screening method such as urine substance testing was not employed. This reliance on self-reporting may have caused a possible underreporting because some respondents could have hesitated to disclose substance use due to legal concerns or fearing how the information would be used leading to desirability biases. A way to overcome this would be to utilize objective measurements for substance use such as urine or blood testing for substances. The low number of participants who had used substances in this study (n = 30) may also lead to issues with generalizability. Furthermore, the internal reliability scores for the questionnaires were below the recommended score and may have limited applicability. Therefore, interpretation of our findings may have limited evidence and future exploration via a qualitative study may help elaborate the study’s results further.

Thirdly, the study only encompassed adolescents between 13 and 16 from a few randomly selected secondary schools in the Northern region of Malaysia, restricting its applicability to other age groups and diminishing the ability to generalize findings to a broader adolescent population nationwide. Additionally, including only Muslim adolescents in the study limits the relevance of the findings to other religious and racial groups in Malaysia, a multicultural country. A diversified sample could provide improved insights into how religiosity, family functioning, and substance use vary across populations. Gender imbalances, with girls outnumbering boys, may have impacted the findings associated with gender differences in substance use. This may affect the generalizability of the findings from this study. The need for parental consent, as the participants were underage, may have introduced self-selection bias because parents with particular views on substance use or religion may have prevented their children’s participation.

## Conclusion and recommendations

Our study revealed that most adolescents who used substances came from low-income families and had parents with low levels of education. Religiosity was not a significant protective factor against substance use. However, being male and having a family history of substance use were significant predictors.

Our simple logistic regression findings demonstrated the importance of having good, healthy family functioning in protecting adolescents from substance use. Thus, family members must be included in the substance use recovery journey for adolescents. Furthermore, we would like to highlight the importance of satisfaction with one’s family and how pivotal it may be in ensuring successful substance use treatment. Two critical takeaways include improving the overall skills constituting a functional family (i.e., communication and problem-solving skills) and enhancing contentment with ones’ family. These two issues could be incorporated into the family-based therapy of adolescents for substance use.

Additionally, special attention should be directed to male adolescents and adolescents with a past family history of substance use because they are at a heightened risk for substance use. It would be beneficial for agencies (government or non-governmental) working with adolescents to contemplate extra attention for these adolescents to protect them from being involved with substances. This may be in the form of improved screening practices for substance use amongst these adolescents during routine medical checkups, promoting better drug education amongst adolescents, or empowering teachers with the knowledge to detect substance use amongst those at risk. These steps would enable not just a timely diagnosis but also a timely treatment intervention for the affected adolescents.

Even though we did not find a significant correlation between religiosity and substance use due to research limitations, past studies have found that religiosity remains an essential factor in influencing substance use. Therefore, exploring the possibilities of interaction between family members, society, peers, and culture regarding religiosity may facilitate identifying the possible influence of religiosity on Malaysian adolescents in substance use cases. Furthermore, it would be beneficial to also assess multiple domains of religiosity as they each may play different roles in their relation to substance use. We would also like to suggest nationwide research in the future covering other possible factors that may play a role in influencing the relationship between religiosity and substance use such as literacy rates, poverty, being in marginalized groups, and past trauma which may aid in the holistic management of adolescent substance use.
